# Supplementary material for: Content-rich biological network constructed by mining PubMed abstracts
Source: BMC Bioinformatics. 2004 Oct 8;5:147. doi: 10.1186/1471-2105-5-147 (PMC528731; doi:10.1186/1471-2105-5-147)
Supplement: Additional File 5 — The original Chilibot query results of the term "long-term potentiation (LTP)" and 22 other terms, limiting the latest references analyzed to the years 1990, 1995, 2000, and 2004. [file 1471-2105-5-147-S5.bz2 › chilibotAdditionalFile5/ltp2004/html/PLC.html]

 


**PLC** (Input: PLC ) 

---


**Google Searches:** Entire Web  | EDU domain only  | PDF files only

.

**External Links:** OMIM |
LocusLink
| Swissprot
| GeneCards

  
**Maps of PLC**

Simple
Complete
graph in
radiant
tree
square
layout.

**New Hypothesis !**

**Synonyms** 

- plc   [PubMed]

**Synopsis**

- These data suggested a bifurcation in the regulation of MAPKs that occurs at the level of **PLC** and RAFTK Pyk2 activation.  OncogeneOncogene, 1996 
  [23]

- These results suggest that two different pathways, the c ras and the **PLC** gamma pathway, are activated by TrkB receptors in primary neurons.  J Neurochem, 1995 
  [23]

- Taken together, our results suggest that a SHPS 1 **PLC** gamma complex activate the **PLC** PKC cascade, which is required for the activation of IL 1beta dependent Erk and Akt signalings and cell proliferation.  Int J Oncol, 2003 
  [20]

- These results taken together suggest that lysophospholipid receptor activation involves receptor coupling to heterotrimeric Gq subunits with consequent activation of **PLC**, PKC, and MAPK pathways leading to ERK phosphorylation.  Glia, 2004 
  [20]

- Cl IB MECA mediated increases in cAMP accumulation suggest that A 3 AR induced ERK1 2 phosphorylation involves adenylyl cyclase activation via phospholipase C **PLC** and PKC stimulation.  Br J Pharmacol, 2004 
  [19]

- Using immunoprecipitation, PI3 kinase and tyrosine phosphorylated **PLC** gamma1 and SHC were shown to be associated with tyrosine phosphorylated TrkB in response to both BDNF and NT 4.  Exp Neurol, 1999 
  [18]

- Finally, inhibition of PI **PLC** results in NGF s ability to stimulate DNA synthesis in the absence and presence of IGF I.  Oncogene, 1999 
  [16]

- We suggest that sustained activation of Trk B  [TRKB]  receptor tyrosine kinase and **PLC** gamma occurs after a brief BDNF application and contributes to the short term maintenance <.  Neuroreport, 2003 
  [16]

- These observations suggest that the 1S,3R ACPD induced burst firing is NOT mediated by **PLC** coupled metabotropic glutamate receptors.  Neuropharmacology, 1994 
  [16]

- These changes in calcium dependent PLA 2 and **PLC** beta 1 activities, coupled with reports of FAE induced deficits in protein kinase C activity, indicate that prenatal exposure to moderate quantities of ethanol causes profound and long lasting deficits in the cellular signaling mechanisms associated with activity dependent synaptic plasticity and memory formation.  Alcohol Clin Exp Res, 1997 
  [16]

- Second, synergy of IP accumulation in correlation with synergy of neurotransmitter release elicited by mAChR activation and membrane depolarization, suggests a possible role for phospholipase C **PLC** in the bifurcating control of neurotransmitter release and for the involvement of **PLC** and voltage sensitive channels in mediation of long term potentiation  [LTP]  LTP .  Neurosci Lett, 1990 
  [16]

- After NGF treatment, NGF was bound to TrkA in endocytic organelles, and TrkA was tyrosine phosphorylated and bound to **PLC** gamma1, suggesting that these receptors were competent to initiate signal transduction.  J Neurosci, 1996 
  [15]

- Recent evidence from this laboratory have demonstrated a critical role of phospholipase A2 PLA2 and arachidonic acid in angiotensin II type 2 AT2 receptor mediated kinase activation in renal epithelium independent of phosphoinositide specific phospholipase C **PLC** and without the necessity of eicosanoid biosynthesis.  Kidney Int, 2004 
  [13]

- The evidence presented is consistent with the hypothesis that **PLC** acts as a coincidence detector in LTP.  Hippocampus, 1998 
  [12]

- Thus, activation of TrkB and **PLC** leads to a TRPC3 dependent cation influx in CNS neurons.  Neuron, 1999 
  [12]
